# Cerebrospinal fluid detection of interleukin-1β in phase of remission predicts disease progression in multiple sclerosis

**DOI:** 10.1186/1742-2094-11-32

**Published:** 2014-02-18

**Authors:** Silvia Rossi, Valeria Studer, Caterina Motta, Giorgio Germani, Giulia Macchiarulo, Fabio Buttari, Raffaele Mancino, Maura Castelli, Valentina De Chiara, Sagit Weiss, Gianvito Martino, Roberto Furlan, Diego Centonze

**Affiliations:** 1 Clinica Neurologica, Dipartimento di Medicina dei Sistemi, Università Tor Vergata, Via Montpellier 1, 00133 Rome, Italy; 2 Fondazione Santa Lucia/Centro Europeo per la Ricerca sul Cervello (CERC), Via del Fosso di Fiorano 64, 00143 Rome, Italy; 3 Clinica Oculistica, Dipartimento di Medicina Sperimentale e Chirurgia, Università Tor Vergata, Via Montpellier 1, 00133 Rome, Italy; 4 Neuroimmunology Unit, Institute of Experimental Neurology (INSpe), Division of Neuroscience, San Raffaele Scientific Institute, Via Olgettina 58, 20132 Milan, Italy

**Keywords:** Cerebrospinal fluid, Cytokines, Inflammation, Disability, Neurodegeneration, Remission

## Abstract

**Background:**

Absence of clinical and radiological activity in relapsing–remitting multiple sclerosis (RRMS) is perceived as disease remission. We explored the role of persisting inflammation during remission in disease evolution.

**Methods:**

Cerebrospinal fluid (CSF) levels of interleukin 1β (IL-1β), a major proinflammatory cytokine, were measured in 170 RRMS patients at the time of clinical and radiological remission. These patients were then followed up for at least 4 years, and clinical, magnetic resonance imaging (MRI) and optical coherence tomography (OCT) measures of disease progression were recorded.

**Results:**

Median follow-up of RRMS patients was 5 years. Detection of CSF IL-1β levels at the time of remission did not predict earlier relapse or new MRI lesion formation. Detection of IL-1β in the CSF was instead associated with higher progression index (PI) and Multiple Sclerosis Severity Scale (MSSS) scores at follow-up, and the number of patients with sustained Expanded Disability Status Scale (EDSS) or Multiple Sclerosis Functional Composite worsening at follow-up was higher in individuals with detectable levels of IL-1β. Patients with undetectable IL-1β in the CSF had significantly lower PI and MSSS scores and a higher probability of having a benign MS phenotype. Furthermore, patients with undetectable CSF levels of IL-1β had less retinal nerve fiber layer thickness and macular volume alterations visualized by OCT compared to patients with detectable IL-1β.

**Conclusions:**

Our results suggest that persistence of a proinflammatory environment in RRMS patients during clinical and radiological remission influences midterm disease progression. Detection of IL-1β in the CSF at the time of remission appears to be a potential negative prognostic factor in RRMS patients.

## Background

Relapsing–remitting multiple sclerosis (RRMS) was originally described as a disease characterized by alternating symptomatic and asymptomatic periods, which were perceived to reflect, respectively, disease activity and remission. With the advent of conventional and, later, unconventional magnetic resonance imaging (MRI) technologies, it appeared that the disease could be active in asymptomatic patients, leading to an extension of diagnostic criteria for RRMS and response to treatment to radiological parameters. Hints suggesting that disability in RRMS patients has additional causes beyond clinical and radiological relapses came from observations in patients with brain atrophy and reduction of *N*-acetyl aspartate, a marker for axonal loss. Brain atrophy appeared to progress in patients who were not active [1], whereas brain atrophy progression [2] and reduction of *N*-acetyl aspartate [3] correlated with disability progression, indicating that disease severity was not determined by relapses only. In line with these observations, researchers who have conducted pathological studies have reported synapse, neuronal and glial loss independent of demyelination [3-9]. Further, investigators have reported evidence of neuronal and glial excitotoxicity in MS [10-12], a central nervous system (CNS)–specific cellular death pathway triggered by an excess of excitatory glutamate signaling. In this respect, we have recently demonstrated that, during MS relapses, cerebrospinal fluid (CSF) concentrations of the proinflammatory cytokine interleukin 1β (IL-1β) increase to a level high enough to boost excitatory transmission and excitotoxic damage in neurons [13]. These premises prompted us to explore whether persistence of IL-1β signaling during remission phases of MS could affect the severity of the disease. In accord with this hypothesis, our results show that detection of the proinflammatory cytokine IL-1β in the CSF of early RRMS patients at the time of remission was associated with pronounced neuronal damage and accumulating disability in the following years.

## Methods

This study was conducted in compliance with the principles of the Declaration of Helsinki and was approved by the Ethical Committee of the Policlinico Università Tor Vergata in Rome. All the participants gave their written informed consent to be included in the study.

### Multiple sclerosis patients and cerebrospinal fluid collection

**Figure 1:**
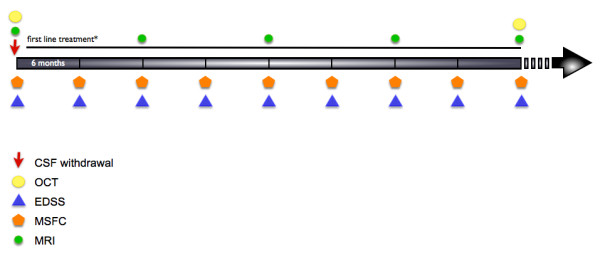
**Study design.** Participants were followed for at least 4 years after cerebrospinal fluid (CSF) collection. Clinical assessment was performed every 6 months, Magnetic resonance imaging (MRI) scans were obtained at least annually. *Second-line treatments were administered to patients who experienced at least two relapses during 1 year of therapy with immunomodulatory agents. EDSS, Expanded Disability Status Scale; MSFC, Multiple Sclerosis Functional Composite; OCT, optical coherence tomography.

A total of 170 central-southern Italian subjects with a diagnosis of RRMS according to the 2005 McDonald criteria [14] were included in this study (Figure 1). CSF was collected at the time of diagnosis from patients in clinical and radiological remission. In particular, patients who had experienced a clinical relapse within the preceding 60 days or had shown gadolinium (Gd)-enhanced lesions or new lesions visualized by T2-weighted MRI were excluded. Relapses were defined as the development of new or recurrent neurological symptoms not associated with fever or infection lasting for at least 24 hours. Lumbar puncture and brain MRI were performed within 24 hours of each other. Blood sample collection, CSF withdrawal and clinical assessments were performed at the MS Center of the Tor Vergata University Hospital of Rome by MS specialist neurologists. To be recruited into the study, patients had to have had at least 4 years of clinical, MRI and optical coherence tomography (OCT) follow-up after CSF collection. In addition, only patients clinically in the remitting phase of MS and without MRI evidence of new or active lesions at the time of CSF withdrawal were considered for inclusion in the present investigation. Accordingly, only transient elevation of proinflammatory cytokines has been reported during MRI or clinical reactivation in MS patients [13,15]. Demographic and clinical information were derived from medical records. MS disease onset was defined as the first episode of focal neurological dysfunction indicative of MS. Disease duration was estimated as the number of years from onset to the most recent assessment of disability. The Bayesian Risk Estimate for Multiple Sclerosis (BREMS) score was calculated using gender, age at onset and clinical events during the first year of disease to identify individual risk of secondary progression [16]. At the time of confirmed diagnosis, all MS patients had started disease-modifying therapy (daily glatiramer acetate 20 mg subcutaneously (s.c.), interferon β-1a (IFN-β-1a) 44 μg s.c. three times weekly, IFN-β-1a 30 μg intramuscularly or IFN-β-1b 250 μg s.c. every other day). Mitoxantrone (12 mg/m^2^ intravenously (i.v.) every 3 months with a lifetime maximum of 140 mg/m^2^), natalizumab (300 mg i.v. every 4 weeks) and fingolimod (0.5 mg by mouth every day) were considered as second-line treatments for patients who experienced at least two relapses during 1 year of therapy with other approved immunomodulatory agents. The annualized relapse rate (ARR) was defined as the number of relapses per year. In addition, the number of relapses in the first 2 years of the disease course and the time until the first relapse were used as clinical indexes of inflammatory activity.

### Disability assessment

Disability was determined by a specially trained (Neurostatus: Available at http://www.neurostatus.net/index.php?file=start) and certified examining neurologist using the Expanded Disability Status Scale (EDSS), a ten-point disease severity score derived from nine ratings for individual neurological domains [17]. The EDSS score, evaluated every 6 months after diagnosis, was used in combination with disease duration to calculate two measures of disease severity: the progression index (PI) and the Multiple Sclerosis Severity Scale (MSSS). The PI was defined as EDSS divided by disease duration. The MSSS is an algorithm that relates EDSS scores to distribution of disability in patients with comparable disease durations [18]. Sustained EDSS progression was defined as a one-point increase persisting for at least 6 months. The Multiple Sclerosis Functional Composite (MSFC) score [19], obtained on the same day as the EDSS score, consists of three domains in separate measurements: scoring ambulation (Timed Walk Test), upper-extremity function (Nine Hole Peg Test) and cognition (Paced Auditory Serial Addition Test). These separate quantitative scores were used in our analyses. Disability progression using the MSFC score was defined as a sustained change of at least 20% from baseline for any of the three components of the MSFC. Our definition of disability progression also required worsening that persisted for at least 6 months. EDSS and MSFC scores were taken into account for the assessment of disability progression when obtained at least 30 days since stabilization and/or resolution of a previous relapse and/or corticosteroid treatment.

### Optical coherence tomography

A medical history with respect to visual symptoms was obtained from all MS participants. Self-report and physician report were confirmed by record review. A subset of RRMS patients (*n* = 118) without a history of optic neuritis and ophthalmological disease underwent measurement of retinal nerve fiber layer (RNFL) thickness and macular volume (MV) for both eyes using Stratus OCT System software version 4.0.2 (Carl Zeiss Meditec, Jena, Germany) [20]. Briefly, for MV, retinal thickness was measured automatically as the distance between the vitreoretinal interface and the anterior boundary of the retinal pigment epithelium. Stratus OCT images were generated using the fast mapping scan protocol, consisting of six radial scans spaced 30° apart, with each scan measuring 6 mm in length. Each image had a resolution of 10 μm axially and 20 μm transversally. All Stratus OCT images had a signal strength of 6 μm. RNFL thickness measurements were read from the automated measurements generated by the machine using Fast RNFL analysis. Scanning was performed after pharmacological dilation. Average RNFL thickness for 360° around the optic disc was recorded. Values were adjusted for age. One randomly chosen eye from each participant was included in the study. Testing was performed by trained technicians experienced in examination of patients for research studies, and patients wore their habitual eyeglasses or contact lenses for distance correction.

### MRI

MRI scans (1.5 T), which consisted of dual-echo proton density, fluid-attenuated inversion recovery, T2-weighted spin-echo images (T2-WI) and pre- and post-contrast-enhanced T1-weighted spin-echo images (T1-WI), were analyzed by a neuroradiologist who was unaware of the patient’s clinical details. A new Gd + (0.2 ml/kg, intravenously) lesion was defined as a typical area of hyperintense signaling on post-contrast-enhanced T1-WI. A new or newly enlarging lesion on T2-WI was defined as a rounded or oval lesion arising from an area previously considered as normal-appearing brain tissue and/or showing an identifiable increase in size from a previously stable-appearing lesion. An active scan was defined as one showing any new, enlarging or recurrent lesions on post-contrast-enhanced T1-WI and T2-WI. T2-WI lesion volume was determined by manual tracing.

### Measurement of IL-1β in cerebrospinal fluid

CSF was centrifuged and immediately stored at −80°C until analyzed using a Bio-Plex multiplex cytokine assay (Bio-Rad Laboratories, Hercules, CA, USA) according to the manufacturer’s instructions. Concentrations of IL-1β (171-A11127; Bio-Rad Laboratories) were calculated according to a standard curve generated for each target and expressed in picograms per milliliter. When the cytokine concentrations were below the detection threshold, they were assumed to be 0 pg/ml.

### Statistical analysis

Participants with MS were divided into two groups according to the detectability (+ group) or undetectability (− group) of IL-1β in the CSF. Differences among groups were compared by univariate analysis using Student’s *t*-test or Mann–Whitney *U* test for continuous variables and Fisher’s exact test or χ^2^ test for categorical variables. Survival curves were analyzed using a logrank (Mantel–Cox) test. Logistic regression models were constructed for the disability as outcome. We estimated the degree of disability by means of the dichotomous EDSS (cutoff point of 3.0 and 4.0, at which, respectively, significant clinical disability and restriction in ambulation start to be appreciated). Four variables (years with disease, age at the time of blood draw, gender and cytokine detection) were included as predictor variables. Disability progression was also assessed by sustained MSFC worsening. The analyses were replicated with the use of second-line treatments taken into consideration as a covariate. In a further model, benign MS status, defined by an EDSS score less than 3.0 15 years or more after disease onset [21], was included as an outcome variable and BREMS score, age and cytokine detection as predictors. Two-way analysis of variance was performed to analyze the main effects of two conditions (cytokine detection versus disease duration) on the dependent variables (ophthalmologic variables) and their interactions. A *P*-value less than 0.05 was considered statistically significant.

## Results

### Patient characteristics

The demographic features and clinical characteristics of RRMS patients are shown in Table 1. The median follow-up duration was 5 years. The minimum and maximum last EDSS values were 0 and 6.5, respectively. All patients had received immunomodulatory treatment during the course of their disease. All of them received first-line treatments since the time of their diagnosis as specified in the Methods section. Some patients (52%) had two immunomodulatory treatments. Patient characteristics according to CSF IL-1β contents are shown in Table 1. The mean EDSS was lower among patients with undetectable IL-1β (*P* < 0.01).

**Table 1:** **Demographic and clinical characteristics of patients with multiple sclerosis according to cerebrospinal fluid proinflammatory cytokine content**^**a**^

**Characteristics**		**IL-1β**		
	**Total**	**+**	−	***P*-value**
Number	170	77	93	
Gender (M/F)	62/108	27/50	35/58	0.75
Age (years)	36.3 ± 9.5	37.8 ± 10.1	35.1 ± 8.8	0.07
Disease duration (years)	10.5 ± 5.3	10.9 ± 5.9	10.2 ± 4.8	0.39
EDSS	2.2 ± 1.7	2.8 ± 1.9	1.7 ± 1.4	< 0.01
BREMS	0.34 ± 0.9	0.47 ± 1.0	0.23 ± 0.8	0.10

^a^+, detectable; −, undetectable; BREMS, Bayesian Risk Estimate for Multiple Sclerosis; EDSS, Expanded Disability Status Scale; F, female; IL-1β, interleukin 1β; M, male. Data are mean ± SD.

### Lack of association between prospective disease activity and cerebrospinal fluid IL-1β level at time of remission

**Figure 2:**
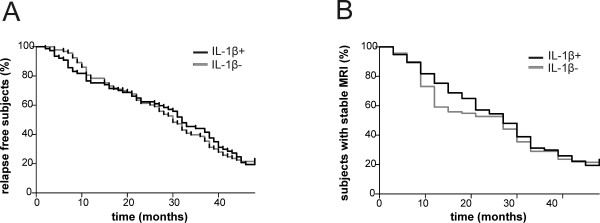
**Interleukin 1β does not influence disease inflammatory activity in relapsing–remitting multiple sclerosis. (A)** and **(B)** Survival analyses for the time to first clinical relapse **(A)** and the time to detecting an active magnetic resonance imaging (MRI) scan since diagnosis **(B)**, among participants with detectable or undetectable levels of interleukin 1β (IL-1β) in the cerebrospinal fluid. No significant differences were observed.

We have previously shown enhanced free IL-1β levels and IL-1β-mediated neurotoxicity in the CSF of patients with active MS and Gd + lesions [13]. In the current study, we analyzed clinical and MRI indexes of inflammatory activity in RRMS patients, whom we stratified by CSF detection of IL-1β during the clinical and radiological remission phase. No significant differences were observed for either examined parameter. In particular, the mean ARR in the first 4 years after diagnosis (IL-1β+: 0.44 ± 0.32 versus IL-1β−: 0.45 ± 0.34), the number of participants with two or more clinical relapses within the first 2 years after the disease diagnosis (IL-1β+: 37.6% versus IL-1β−: 38.7%), the number of participants with an MRI scan showing active MS within the first 2 years after the disease diagnosis (IL-1β+: 45.4% versus IL-1β−: 44.9%), the number of patients prescribed a second-line treatment (IL-1β+: 28.5% versus IL-1β−: 26.8%) and T2-WI-detected lesion volume (IL-1β+: 8,741.8 ± 2,674.5 mm^3^ versus IL-1β−: 8,486.4 ± 2,903.9 mm^3^) were similar (*P* > 0.05 for each comparison). In line with these findings, no significant differences between the groups were revealed by survival analysis for time to first clinical relapse (*P* > 0.05) (Figure 2A) and the time to detection of an active MRI scan since diagnosis (*P* > 0.05) (Figure 2B).

### Association between prospective disease progression and cerebrospinal fluid IL-1β detection at time of remission

**Figure 3:**
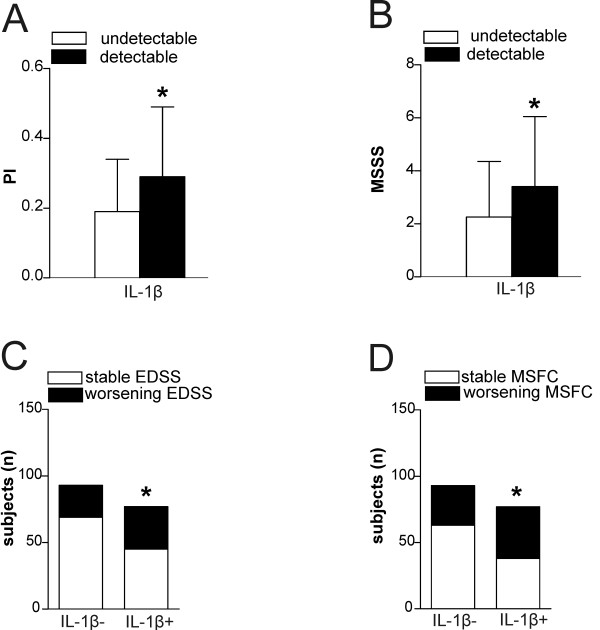
**Interleukin 1β influences disease progression in relapsing–remitting multiple sclerosis. (A)** Progression index (PI) and **(B)** Multiple Sclerosis Severity Scale (MSSS) scores were higher among participants with detectable interleukin 1β (IL-1β) in the cerebrospinal fluid. Disability progression, measured as a sustained change in score on the Expanded Disability Status Scale (EDSS) **(C)** or Multiple Sclerosis Functional Composite (MSFC) **(D)**, was higher among participants with detectable IL-1β. *P < 0.05.

When we compared patients with undetectable vs. those with detectable CSF IL-1β levels at baseline, we found that mean PI and MSSS scores were significantly lower among participants with undetectable IL-1β (*P* < 0.01 for both parameters) (Figures 3A and 3B). The number of participants with sustained worsening according to EDSS or MSFC score at follow-up was higher in the IL-1β + group (EDSS: 41.5% versus 25.8%, *P* = 0.03; MSFC: 50.6% versus 32.2%, *P* = 0.02) (Figures 3C and 3D). By applying multiple logistic regression analysis with dichotomous EDSS score as the response variable and the variables described in the Methods section as predictors, the probability of reaching an EDSS score of 3.0 at follow-up was significantly affected by the presence of IL-1β in CSF and by the duration of the disease (Table 2). The analysis replicated using an EDSS score of 4.0 as the cutoff confirmed the association between IL-1β detection in CSF in patients in remission and the probability of reaching higher EDSS values at the follow-up examination (Table 3). Similar results were obtained when MSFC worsening was included as an outcome variable. In fact, the logistic regression predicted that, at equal values for age, gender and disease duration, the probability of disability progression according to MSFC score increased in the IL-1β + group (coefficient of correlation: 0.75, SE: 0.33, odds ratio (OR): 2.13, 95% confidence interval (CI): 1.11 to 4.09, *P* = 0.02). The analyses were replicated taking into consideration the use of second-line treatments as a covariate. In addition, using the same model, the presence of IL-1β in CSF significantly affected the probability of reaching an EDSS score of 3.0 (coefficient of correlation: 1.47, SE: 0.42, OR: 4.34, 95% CI: 1.93 to 9.83, *P* < 0.001), EDSS score of 4.0 (coefficient of correlation: 1.41, SE: 0.45, OR: 4.12, 95% CI: 1.69 to 10.02, *P* = 0.001) and MSFC worsening (coefficient of correlation: 0.79, SE: 0.35, OR: 2.21, 95% CI: 1.10 to 4.41, *P* = 0.02).

**Table 2:** **Logistic regression data using Expanding Disability Status Scale score ≥3.0 as the response variable**^**a**^

**Predictor variable**	**Coefficient of correlation**	**SE**	**OR**	**95% confidence interval**	***P*-value**
IL-1β detection	1.22	0.35	3.38	1.69 to 6.79	<0.001
Age	0.01	0.02	1.01	0.97 to 1.05	0.59
Gender (M)	0.64	0.36	1.90	0.93 to 3.87	0.07
Disease duration	0.09	0.03	1.09	1.02 to 1.17	0.01

^a^IL-1β, interleukin 1β; OR, odds ratio; SE, standard error.

**Table 3:** **Logistic regression using Expanding Disability Status Scale score ≥4.0 as the response variable**^**a**^

**Predictor variable**	**Coefficient of correlation**	**SE**	**OR**	**95% confidence interval**	***P*-value**
IL-1β detection	1.20	0.39	3.32	1.54 to 7.19	0.002
Age	0.02	0.02	1.02	0.98 to 1.07	0.30
Gender (M)	0.49	0.39	1.63	0.75 to 3.50	0.20
Disease duration	0.05	0.03	1.05	0.98 to 1.13	0.15

^a^IL-1β, interleukin 1β; M, male; OR, odds ratio; SE, standard error.

Patients with high BREMS scores are considered at very high risk of secondary progression, and patients with low BREMS scores are very likely to remain progression-free [16]. Undetectable IL-1β significantly predicted benign MS at equal BREMS score values (coefficient of correlation: 3.28, SE: 1.51, OR: 26.61, 95% CI: 1.37 to 515.47, *P* = 0.03).

### Association between cerebrospinal fluid IL-1β level detection and neuronal damage

**Figure 4:**
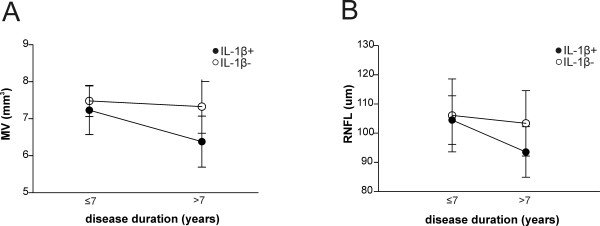
**Interleukin 1β influences neuronal damage in RR multiple sclerosis.** Plots of interaction analysis between interleukin 1β (IL-1β) detection in the cerebrospinal fluid and disease duration, analysis of optical coherence tomography parameters, macular volume (MV) **(A)** and retinal nerve fiber layer thickness (RNFL) thickness **(B)**. These data confirm the relative preservation of neuronal structures among the participants with undetectable IL-1β, despite disease duration.

We also investigated the possible relationship between CSF IL-1β content at the time of the clinical and radiological remission phase at baseline and OCT parameters at follow-up in RRMS patients with the same disease duration (disease duration ≤7 years: IL-1β + (*n* = 23), IL-1β − (*n* = 23); disease duration >7 years: IL-1β + (*n* = 33), IL-1β − (*n* = 39)). A significant main effect of CSF IL-1β content was revealed by analyzing both RNFL thickness (F = 8.08, *P* = 0.005) and MV (F = 23.74, *P* < 0.0001), indicating damage to axonal and neuronal structures among the IL-1β + group. Disease duration also affected OCT parameters (RNFL thickness: F = 11.50, *P* = 0.001; MV: F = 16.48, *P* = 0.0001), with a significant interaction with IL-1β detection (RNFL thickness: F = 4.46, *P* = 0.03; MV: F = 8.07, *P* = 0.005), confirming less severe neurodegenerative damage in participants with undetectable IL-1β in spite of longer disease duration (Figures 4A and 4B).

## Discussion

Our aim in the present study was to explore the role of silent inflammation in MS pathophysiology by studying the correlation between CSF IL-1β content at the time of clinical and radiological remission with markers of disease activity, disability progression and neuronal damage [22,23] at midterm follow-up. To the best of our knowledge, herein we report for the first time that the presence of IL-1β in the CSF of RRMS patients at the time of clinical and radiological remission was associated with disability progression and neuronal damage after a median of 5 years of follow-up. Our data show that patients with undetectable CSF levels of IL-1β had a high probability of remaining at low levels of disability, whereas patients with detectable IL-1β had a higher risk for progression of disability and greater restriction in ambulation as measured by both EDSS and MSFC scores. In fact, the probability of reaching an EDSS score of 3 or 4 during follow-up was lower in patients with undetectable IL-1β, and undetectable IL-1β significantly predicted benign MS, at equal BREMS scores. Further studies are warranted to confirm our results by means of quantitative PCR. In fact, a more precise cutoff of CSF IL-1β levels below the detection threshold of the immunosorbent assay that we performed could be useful to better identify progression-free patients.

Our OCT measurements of neuronal and axonal damage were in line with these data, as patients with undetectable IL-1β had higher values of RNFL thickness and MV. CSF IL-1β level influenced the risk for disability progression without impacting clinical and radiological markers of inflammatory activity. These data shed new light on our understanding of the disease mechanisms in MS. They suggest that in fact a complete resolution of inflammation after a relapse, as reflected by undetectable presence of IL-1β in the CSF, is potentially a determining factor in midterm disability and prognosis for patients with MS. Persistent inflammation during clinical remission has been previously reported. Overexpression of genes related to adhesion, chemotaxis and blood–brain barrier damage, such as matrix metalloproteinase 9, chemokine (C-C motif) ligand 19 and intercellular adhesion molecule 5, has been described in patients with remitting MS, suggesting persistent inflammation during clinical remission [24]. In line with this observation, the percentage of CD4 + tumor necrosis factor α-positive-IL-2− T cells in the CSF of RRMS patients in clinical remission was increased compared with CSF from patients with noninflammatory disease [15]. Our data support the idea that persisting inflammation during clinical and radiological remission is not just a remnant of the relapse-associated acute inflammation, but is a damaging phenomenon with the potential to lead to significant clinical midterm disease outcomes. Patients with undetectable IL-1β in fact had a higher probability of presenting with a benign MS phenotype. Thus, we hypothesize that when immune challenge becomes chronic instead of being transient, the CNS is chronically exposed to cytokines with maladaptive effects such as neuronal and axonal dysfunction evolving to irreversible damage and sustained neurological disability. Long-term disability progression in patients with relapse-onset MS is correlated with degree of disability after 5 years [25], relapse frequency and interval between relapses during the first 2 years [26] and incomplete recovery from relapses during the first 5 years after onset [27]. Nonclinical early predictors of long-term disability have also been identified: brain atrophy rate [28-31], baseline lesion volume [31,32] and long-term increase in lesion volume [30-33]. More recently, long-term disability has also been correlated to measures of gray matter atrophy [34-36] and altered evoked potentials [37-39]. Further research is needed to clarify the source of proinflammatory cytokines during apparent clinical and radiological remission to better define the role of smoldering inflammation in long-term accumulation of neuronal damage and disability.

## Conclusions

We propose that predicting a long-term good prognosis in RRMS would require evidence for complete inflammation resolution during remission in addition to the criteria of disease-free status as recently defined [40].

## Competing interests

SR received honoraria for writing from Bayer Schering and funding for traveling from Novartis, Teva and Merck Serono. She acted as an advisory board member of Biogen Idec and is involved as a subinvestigator in clinical trials for Novartis, Merck Serono, Teva, Bayer Schering, Sanofi-aventis, Biogen Idec and Roche. VDC received funding for traveling by Teva. She is involved as study coordinator in clinical trials for Novartis, Merck Serono, Teva, Bayer Schering, Sanofi-aventis, Biogen Idec and Roche. DC served as an advisory board member of Merck-Serono, Teva, Bayer Schering, Biogen Idec, Novartis and Almirall and received funding for travel, honoraria for speaking or consultation fees from Merck Serono, Teva, Novartis, Bayer Schering, Sanofi-aventis and Biogen Idec. He is the Principal Investigator in clinical trials for Novartis, Merck Serono, Teva, Bayer Schering, Sanofi-aventis, Biogen Idec and Roche. The other authors declare that they have no competing interests.

## Authors’ contributions

SR and DC were involved in study concept and design. VS, CM, GG, GM, FB, RM, MC and VD were involved in the acquisition of data. SR, SW and DC analyzed and interpreted data. SR and SW drafted the manuscript. Statistical analysis was performed by SR. DC critically revised the manuscript for important intellectual content. All authors read and approved the final manuscript.
